# Supplementary material for: Knockdown of the Halloween Genes spook, shadow and shade Influences Oocyte Development, Egg Shape, Oviposition and Hatching in the Desert Locust
Source: Int J Mol Sci. 2022 Aug 17;23(16):9232. doi: 10.3390/ijms23169232 (PMC9408901; doi:10.3390/ijms23169232)
Supplement: Supplementary file 1 [file ijms-23-09232-s001.zip › ijms-1840943-supplementary.pdf]

International Journal of
Molecular Sciences

MDPI

Article Supplementary file

Knockdown of the *Halloween* genes *spook*, *shadow* and *shade* influences oocyte development, egg shape, oviposition and hatching in the desert locust.

Sam Schellens [1,‡], Cynthia Lenaerts [1,‡], María del Rocío Pérez Baca [1,2], Dorien Cools [1], Paulien Peeters [1], Elisabeth Marchal [1,3] and Jozef Vanden Broeck [1,*]

[1] Molecular Developmental Physiology and Signal Transduction, KU Leuven, Naamsestraat 59 Box 2465, B-3000 Leuven, Belgium.; sam.schellens@kuleuven.be, cynthia.lenaerts@kuleuven.be, dorien.cools@kuleuven.be, paulien.peeters@kuleuven.be, jozef.vandenbroeck@kuleuven.be
[2] Current address: Center of Medical Genetics Ghent, UZ Ghent, Corneel Heymanslaan 10, entrance 34, B-9000 Ghent, Belgium ; MariadelRocio.PerezBaca@UGent.be
[3] Current address: Department of Life Science Technologies, Imec, Kapeldreef 75, B-3001 Leuven, Belgium; Elisabeth.Marchal@imec.be
‡ Equally contributing authors
* Correspondence: jozef.vandenbroeck@kuleuven.be

Abstract: Ecdysteroids are widely investigated for their role during the molting cascade in insects; however, they are also involved in the development of the female reproductive system. Ecdysteroids are synthesized from cholesterol, which is further converted via a series of enzymatic steps into the main molting hormone, 20-hydoxyecdysone. Most of these biosynthetic conversion steps involve the activity of cytochrome P450 (CYP) hydroxylases, which are encoded by the *Halloween* genes. Three of these genes, *spook* (*spo*), *phantom* (*phm*) and *shade* (*shd*), were previously characterized in the desert locust, *Schistocerca gregaria*. Based on recent sequencing data, we have now identified the sequences of *disembodied* (*dib*) and *shadow* (*sad*), for which we also analyzed spatiotemporal expression profiles using qRT-PCR. Furthermore, we investigated the possible role(s) of five different *Halloween* genes in the oogenesis process by means of RNA interference mediated knockdown experiments. Our results showed that depleting the expression of *SchgrSpo*, *SchgrSad* and *SchgrShd* had a significant impact on oocyte development, oviposition and hatching of the eggs. Moreover, the shape of the growing oocytes, as well as the deposited eggs, was very drastically altered by the experimental treatments. Consequently, it can be proposed that these three enzymes play an important role in oogenesis.

Keywords: Biosynthesis; Ecdysone; Ecdysteriod; Hemimetabola; Hormone; Insect; Oogenesis; Reproduction

Citation: Lastname, F.; Lastname, F.; Lastname, F. Title. *Int. J. Mol. Sci.* **2022**, *23*, 9232. https://doi.org/10.3390/ijms23169232

Academic Editor: Firstname Lastname

Received: date
Accepted: date
Published: date

Publisher's Note: MDPI stays neutral with regard to jurisdictional claims in published maps and institutional affiliations.

Copyright: © 2022 by the authors. Submitted for possible open access publication under the terms and conditions of the Creative Commons Attribution (CC BY) license (https://creativecommons.org/licenses/by/4.0/).

1. Supplementary data

ATG	AGA	AAT	TAC	CAT	TGT	CCC	AGG	CTG	CTA	TCG	AGA	CTG	AAA	TTG	GAC
M	R	N	Y	H	C	P	R	L	L	S	R	L	K	L	D
GAA	TTT	GAG	AAA	CTA	CAT	AGG	CCT	GGT	CGT	CGT	CTA	TTT	CAG	AAG	TGT
E	F	E	K	L	H	R	P	G	R	R	L	F	Q	K	C
TAC	AGC	ACA	CAT	AGC	ACT	GTA	ACA	ACT	GGA	ACA	TCA	GAC	CCC	AAA	TTA
Y	S	T	H	S	T	V	T	T	G	T	S	D	P	K	L
TTT	AAA	CAC	ATA	CCT	GGA	CCC	AAA	TCA	CTA	CCA	CTG	ATT	GGA	ACT	TTG
F	K	H	I	P	G	P	K	S	L	P	L	I	G	T	L
TTA	AAT	TAT	GTT	CCA	TTA	CTT	GGT	GAC	TAT	AAG	TTT	GAT	AGA	TTG	CAT
L	N	Y	V	P	L	L	G	D	Y	K	F	D	R	L	H
CGA	AAT	GGA	ATG	AAG	AAG	TTG	AAG	AAA	TAT	GGA	AAG	CTT	GTG	AAA	GAA
R	N	G	M	K	K	L	K	K	Y	G	K	L	V	K	E
GAA	ATT	GTA	CCT	GGT	GTT	AAT	GTT	GTA	TGG	GTC	TTT	GAC	CCA	TCT	GAT
E	I	V	P	G	V	N	V	V	W	V	F	D	P	S	D
ATT	GAA	ACA	GTA	TTC	AAA	AGT	GAA	GGA	CGA	TAT	CCA	GAG	CGC	CGA	AGT
I	E	T	V	F	K	S	E	G	R	Y	P	E	R	R	S
CAT	CTT	GCA	GTG	GAA	AAG	TAC	CGA	CGA	GAG	AGA	CCT	GAA	ATC	TAC	AGC
H	L	A	V	E	K	Y	R	R	E	R	P	E	I	Y	S
AAT	GGT	GGT	CTT	TTG	CCT	ACG	AAT	GGA	GCA	GAT	TGG	TGG	CAT	CTG	AGA
N	G	G	L	L	P	T	N	G	A	D	W	W	H	L	R
AGT	AAA	TTT	CAG	CAA	GCA	TTG	AGT	CGA	CCC	CAA	AAT	GTT	AAG	CTG	TAC
S	K	F	Q	Q	A	L	S	R	P	Q	N	V	K	L	Y
ATA	GAA	CAA	ACT	GAT	GCT	GTA	ATT	CAA	GAA	TTT	ATA	GAA	CTG	ATC	AGA
I	E	Q	T	D	A	V	I	Q	E	F	I	E	L	I	R
AGA	CAT	TCA	AAA	TCT	CAA	AAA	CCA	CAT	GAT	TAT	CTA	CAT	GAT	TTG	TCT
R	H	S	K	S	Q	K	P	H	D	Y	L	H	D	L	S
CAT	CTT	TTC	ATG	GAA	TTG	GTG	GGG	TTA	GTG	GCA	TTT	GAC	GTT	AAT	TTA
H	L	F	M	E	L	V	G	L	V	A	F	D	V	N	L
GGC	AGC	CTT	ACT	GAA	GAT	GGA	CTG	AAA	AAT	GAC	TCT	CGA	TCA	TCA	AAA
G	S	L	T	E	D	G	L	K	N	D	S	R	S	S	K
CTG	ATA	ATT	GCT	GCA	GAG	ACC	ACT	AAC	AGT	TGT	GTT	GTG	GAA	CTA	GAC
L	I	I	A	A	E	T	T	N	S	C	V	V	E	L	D
AAT	GGA	TTA	CAG	CTG	TGG	AAA	TTC	ATG	GAA	ACT	CCA	CTG	TAC	AAG	AAG
N	G	L	Q	L	W	K	F	M	E	T	P	L	Y	K	K
TTC	AAG	ATA	GCA	TAT	CAT	TTT	ATG	GAG	AGT	GTT	GCA	TTG	GAG	CTG	GTA
F	K	I	A	Y	H	F	M	E	S	V	A	L	E	L	V
TCA	CAG	AAA	GCT	ACA	GCT	ATG	GAA	AAA	GGA	TGG	AAG	CGA	AAT	GAT	GGA
S	Q	K	A	T	A	M	E	K	G	W	K	R	N	D	G
CAT	CAG	GCA	TCT	CTT	CTA	GAA	CAG	TAT	CTC	TTA	TCA	CCG	GAT	TTA	AGC
H	Q	A	S	L	L	E	Q	Y	L	L	S	P	D	L	S
CAT	AAG	GAT	ATT	GTA	GGG	ATG	GCA	GTT	GAC	ATG	ATT	TTA	GCT	GGA	ATG
H	K	D	I	V	G	M	A	V	D	M	I	L	A	G	M
GAC	ACA	ACA	TCT	TAT	TCA	TCA	TCA	TTT	GCC	CTG	TAC	CAT	CTA	GCT	ATG
D	T	T	S	Y	S	S	S	F	A	L	Y	H	L	A	M
AAC	CCA	GAA	AGC	CAG	AAG	CTC	ATG	CAT	CAA	GAG	GCA	CTA	AAA	CTT	CTT
N	P	E	S	Q	K	L	M	H	Q	E	A	L	K	L	L
CCA	GAA	AAG	AGT	ACT	CCT	GTA	ACT	TCA	CAA	GTG	CTT	AAT	CAG	GCA	TCC
P	E	K	S	T	P	V	T	S	Q	V	L	N	Q	A	S
TAT	ACA	AAG	GCT	GCC	TTA	AAG	GAG	ACA	TTT	CGT	CTC	AAT	CCA	ATT	TCT
Y	T	K	A	A	L	K	E	T	F	R	L	N	P	I	S
GTA	GGA	ATT	GGG	AGG	ATA	CTG	GCA	CAT	GAT	GCT	GTT	CTT	TCT	GGA	TTC
V	G	I	G	R	I	L	A	H	D	A	V	L	S	G	F
CAT	GTA	CCA	GCA	GGG	ACT	GTG	GTT	GTG	ACA	CAA	AAT	CAA	GTA	TCA	TGT
H	V	P	A	G	T	V	V	V	T	Q	N	Q	V	S	C
CGG	CTA	CCA	GAG	TAT	TTC	CCA	GAC	CCA	GAT	CGC	TTT	GTC	CCA	GAA	AGG
R	L	P	E	Y	F	P	D	P	D	R	F	V	P	E	R
TGG	CTG	AAA	GGA	CAT	GCT	TTG	TAT	CAT	GGT	AGT	GTC	AGT	CCA	TAC	CTG
W	L	K	G	H	A	L	Y	H	G	S	V	S	P	Y	L
GTA	CTT	CCT	TTT	GGG	CAT	GGA	CCA	CGC	AGC	TGT	ATT	GCT	CGC	CGC	CTA
V	L	P	F	G	H	G	P	R	S	C	I	A	R	R	L
GCA	GAG	CAA	AAT	ATG	CAA	ATT	CTG	TTG	CTG	AAG	GTC	TGT	CGT	AAC	TTC
A	E	Q	N	M	Q	I	L	L	L	K	V	C	R	N	F
AAG	ATA	GGC	TGG	TGT	GGT	GGA	CAT	TTG	GAT	GTT	AGG	ACC	CAG	CCA	GTA
K	I	G	W	C	G	G	H	L	D	V	R	T	Q	P	V
AAT	AAG	CCT	GAT	GAA	CCT	GTA	TTA	TTG	ACG	TTT	GAT	GAA	TAT	TGA	
N	K	P	D	E	P	V	L	L	T	F	D	E	Y	*	

Supplementary Figure S1. ***SchgrDib*** **nucleotide sequence and translation.** qPCR primers and primers for generating two dsRNA constructs are indicated in red, blue and green, respectively.

ATG	TGG	AGC	CGT	GTG	CGC	GTG	TGG	TGT	CGC	GGT	TCG	TCG	TGG	GCG	GCG
M	W	S	R	V	R	V	W	C	R	G	S	S	W	A	A
CGC	GCG	CTG	CCC	CGC	CAG	CAG	CTG	CCG	CCC	CCG	CCG	CCG	TCG	CCT	CGC
R	A	L	P	R	Q	Q	L	P	P	P	P	P	S	P	R
GGC	CTG	CCG	CTC	CTC	GGC	ACC	ACG	CTG	GAC	CTG	CTG	GCC	GCC	GGC	GCC
G	L	P	L	L	G	T	T	L	D	L	L	A	A	G	A
GCC	CCC	CGC	CTG	CAC	CGC	TAC	GTC	GAC	CGC	CGG	CAC	CGC	GAG	CTG	GGG
A	P	R	L	H	R	Y	V	D	R	R	H	R	E	L	G
CCC	GTC	TTC	CGG	GAA	TCC	ATA	GGG	CCC	GTC	GAC	GCC	GTC	TTC	GTC	GCC
P	V	F	R	E	S	I	G	P	V	D	A	V	F	V	A
GAC	GCC	AAC	GAG	ATG	CGG	CGC	GTC	TTC	TCC	GTC	GAG	GGG	CGC	TAC	CCC
D	A	N	E	M	R	R	V	F	S	V	E	G	R	Y	P
GCC	CAC	ATC	GTG	CCC	GAG	TCC	TGG	CTG	CTC	TAC	AAC	GAG	GTC	TAC	GGC
A	H	I	V	P	E	S	W	L	L	Y	N	E	V	Y	G
TAC	AAG	CGG	GGG	CTC	TTC	TTC	ATG	GAT	GGC	GAG	GAG	TGG	CTG	CGC	TTC
Y	K	R	G	L	F	F	M	D	G	E	E	W	L	R	F
CGG	CGC	GTG	CTG	AGC	GGG	CAG	CTG	CTG	CGA	GCC	GGC	AGC	GGG	CCC	GAG
R	R	V	L	S	G	Q	L	L	R	A	G	S	G	P	E
CTG	CTG	GTG	GCG	CCG	TGC	CGC	GCC	GTC	GCC	GAG	AAC	TTC	GCC	GCC	GAG
L	L	V	A	P	C	R	A	V	A	E	N	F	A	A	E
CTG	GCG	GCC	GGC	CAG	GTG	GTG	CCC	GAC	CTC	GAG	GGC	CAC	ATG	TAC	CTC
L	A	A	G	Q	V	V	P	D	L	E	G	H	M	Y	L
TGG	GCC	ACC	GAC	GTG	CTG	GTG	GCG	GTG	CTG	CTG	GGG	CAG	AGG	CAG	TAC
W	A	T	D	V	L	V	A	V	L	L	G	Q	R	Q	Y
TCG	GCG	ATG	CGC	TCG	GAG	CTG	CGG	GCG	CCG	ACG	CAA	CAG	CTG	GCG	GCG
S	A	M	R	S	E	L	R	A	P	T	Q	Q	L	A	A
GCC	GTG	CAC	CGC	GTC	TTC	GGC	GAG	TCT	GCG	CGC	CTC	TCT	CTG	CTG	CCG
A	V	H	R	V	F	G	E	S	A	R	L	S	L	L	P
GCG	CGG	CTG	GCG	CGG	CTG	CTG	CGC	CTG	CCC	GCC	TGG	AGG	GAC	TTC	CTG
A	R	L	A	R	L	L	R	L	P	A	W	R	D	F	V
GAC	GCC	GTC	GGC	GCC	GCC	CTC	ACT	CAG	GGT	AAC	GCG	CTG	GTG	ACG	CAG
D	A	V	G	A	A	L	T	Q	G	N	A	L	V	T	Q
CTG	ATC	CCG	GCG	TGC	GAG	GGC	GGC	GCC	GGG	CTG	CTG	GGG	CGG	CTC	CTG
L	I	P	A	C	E	G	G	A	G	L	L	G	R	L	L
GCC	GCC	GGC	GTC	TCC	CAC	GAC	GAC	GCG	CGC	CGC	CTC	GTC	GTC	GAC	CTG
A	A	G	V	S	H	D	D	A	R	R	L	V	V	D	L
CTG	CTC	GCC	GCC	GGA	GAC	ACC	ACG	GCG	TAC	TCG	ACG	CTG	TGG	CTG	CTG
L	L	A	A	G	D	T	T	A	Y	S	T	L	W	L	L
TAC	CTG	CTG	GCG	AAG	CAC	CCG	TCG	GTG	CAG	GAG	CAG	CTG	CAC	CGG	GAG
Y	L	L	A	K	H	P	S	V	Q	E	Q	L	H	R	E
CTG	ACG	CGG	GCG	CCG	GCG	CTG	GCC	GCG	GAG	GAG	GTG	GCG	CGG	CTG	CCG
L	T	R	A	P	A	L	A	A	E	E	V	A	R	L	P
CTG	GCC	AGG	GGC	GCC	ATG	CGG	GAG	GCC	CTC	CGC	CTC	TTC	CCC	GTC	GCG
L	A	R	G	A	M	R	E	A	L	R	L	F	P	V	A
CCC	TTC	CTG	TCG	CGA	TAC	CTG	CCC	GAG	GAC	GCC	ACC	ATC	GGC	GGC	TAC
P	F	L	S	R	Y	L	P	E	D	A	T	I	G	G	Y
CAC	GTC	CCG	GCC	GGG	CAA	CTG	GTG	GTG	TTG	TCG	CTG	TAC	ACG	AGC	GGC
H	V	P	A	G	Q	L	V	V	L	S	L	Y	T	S	G
CGC	AGC	GAG	GAC	TAC	TTC	CCG	CAG	GCG	GGC	CGC	TTC	TGG	CCG	CAG	CGC
R	S	E	D	Y	F	P	Q	A	G	R	F	W	P	Q	R
TGG	CTG	CGG	GAC	CCC	GGG	GGC	GGC	TAC	CGC	GGC	GTC	GCC	GCC	AGC	CAC
W	L	R	D	P	G	G	G	Y	R	G	V	A	A	S	H
GCC	TCG	CTG	CCC	TTC	GCC	ATG	GGC	GCG	CGC	TCC	TGC	GTC	GGC	CAG	AAG
A	S	L	P	F	A	M	G	A	R	S	C	V	G	Q	K
CTC	GCC	GAG	ACG	CAG	ATG	GCG	CTC	ATC	GTG	GCC	GAG	ATT	ACG	AAG	AGG
L	A	E	T	Q	M	A	L	I	V	A	E	I	T	K	R
TTC	CGG	CTG	GAG	CTG	GCC	GAC	GAC	GAG	GAT	CCG	GAG	ATG	GTG	CTG	AGG
F	R	L	E	L	A	D	D	E	D	P	E	M	V	L	R
CTG	GTG	TCG	GTG	CCG	TCG	AAG	CCC	ATC	AGG	CTG	AGG	CCG	GTG	CCC	CGG
L	V	S	V	P	S	K	P	I	E	L	R	P	V	P	R
CAC	TGA														
H	*														

Supplementary Figure S2. ***SchgrSad*** **nucleotide sequence and translation.** qPCR primers and primers for generating two dsRNA constructs are indicated in red, blue and green, respectively.

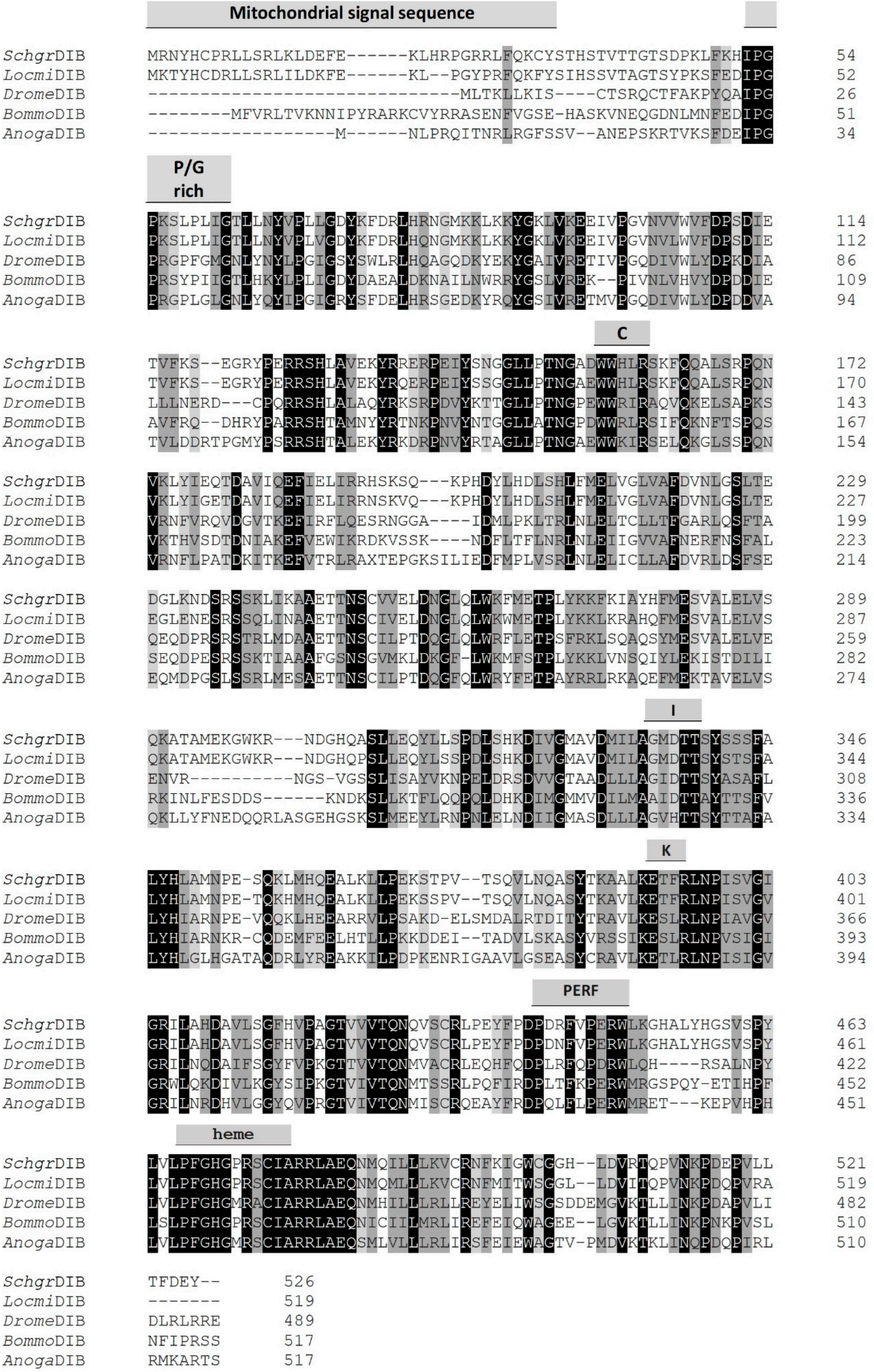

Supplementary Figure S3. Multiple sequence alignment of the cloned *Schgr*DIB (KY404120) with other identified DIB proteins from *Locusta migratoria* (*Locmi*DIB, AVL92819.1), *Drosophila melanogaster* (*Drome*DIB, AAF47831.2),

***Bombyx mori* (*Bommo*DIB, BAD99022.1) and *Anopheles gambiae* (*Anoga*DIB, ABU42523).** The multiple sequence alignment was made using the M-Coffee web server in the default settings (http://tcoffee.crg.cat/apps/tcoffee/do:mcoffee). The degree of conservation was highlighted using Boxshade and is indicated as follows: residues highlighted in black: complete; residues highlighted in dark grey: strong; residues highlighted in light grey: weak. Several key motifs for CYP450 enzymes are shown: mitochondrial signal sequence, P/G rich domain, helix-C, helix-I, helix-K, PERF-motif and the heme-binding domain.

```
SchgrSAD      -----------MWSR---------------VRVWCRG---SSWAARALPR---QQLPPPP      28
LocmiSAD      -----------MWSR---------------AREWSRA---SSWAARALQPQTQQQQLPPP      31
DromeSAD      MTEKRERPGPLRWLRHLLDQLLVRILSLSLFRSRCDPPPLQRFPATELPPAVA-AKYVPI      59
BommoSAD      ---------------------MHRFPSMSSIRSAVRSRNSNRCS-MSTKPHKSLRTIDEM      38
AnogaSAD      --------------------MLQR-LRGTGSRFRCTGGQQQRAG--VAGDSRGAKPFAEM      37

              P/G rich
SchgrSAD      PSPRGLPLLGTTLDLLAAGAAPRLHRYVDRRHRELGPVFRESIGP-VDAVFVADANEMRR      87
LocmiSAD      PAPRGLPLVGTTLDLIAAGSAPRLHHYVDQRHRQLGPVFRDSIGP-VDAVFVSDPNEMRR      90
DromeSAD      PRVKGLPVVGTLVDLIAAGGATHLHKYIDARHKQYGPIFRERLGGTQDAVFVSSANLMRG      119
BommoSAD      PHKKSLPIIGTKFDLFSAGGGKNLHKYIDMRHKQLGPIFYERLTGKTKLVFISDPTHMKS      98
AnogaSAD      PGPRRIPLLGMLNDVMQLGKPAELHLRISKYHEQYGDLVRLQIGT-QNAVFVRDPSLMRK      96

                                                  C
SchgrSAD      VF-SVEGRYPAHIVPESWLLYNEVYGYKRGLFFMDGEEWLRFRRVLSGQLLRAGS-G-PE      144
LocmiSAD      VF-SLEGRYPAHIVPEAWLLYNQVYGYKRGLFFMEGEEWQRFRRVLAGQLLRASS-V-PE      147
DromeSAD      VF-QHEGQYPQHPLPDAWTLYNQQHACQRGLFFMEGAEWLHNRRILNRLLLNGNL-NWMD      177
BommoSAD      LFLNLEGKYPAHILPEPWVLYEKLYGSKRGLFFMDGEDWLINRRIMNKHLLREDSDVWLR      158
AnogaSAD      TF-QLEGAYPRHPLPESWTYFNKKHDYQRGLFFMDGKEWLQSRQIFNKPMLKDF--HWME      153

SchgrSAD      LLVAPCRAVA-ENF--------AAE-----LAAGQVVPDLEGHMYLWATDVLVAVLLGQR      190
LocmiSAD      LLLAPCHAVA-KDF--------AAE-----LAPGQVVPDLESRMYLWATDVLVAVLLGAQ      193
DromeSAD      VHIESCTRRMVDQWKRRTAEAAAIPLAESGEIRSYELPLLEQQLYRWSIEVLCCIMFGTS      237
BommoSAD      APIRTAVFHFICNWKLR-------------AQSGNFSPNLESEFYRFSTDVILAVLQGNS      205
AnogaSAD      EPIRGTCEATVGHMQQT-------------CDSAAAFEGIEAFLYQWSVEVVLSVMLGSA      200

SchgrSAD      QYSAMR-SELRAPTQQLAAAVHRVFGESARLSLLPARLARLLRLPAWRDFVDAVGAALTQ      249
LocmiSAD      RYGELR-SQLRAPTRELAGAVHKVFGESARLSLMPAKLARLLHLPAWRHFVSAVQAALDQ      252
DromeSAD      VLTCP---KIQSSLDYFTQIVHKVFEHSSRLMTFPPRLAQILRLPIWRDFEANVDEVLRE      294
BommoSAD      ALLKPT-PEYEMLLLLFSEAVKKIFSTTTKLYALPVEFCQRWNLKVWRNFKQSVDDSISI      264
AnogaSAD      FTECQQSAEFRRLVQQFSAVVYDIFRCSSELMNIPPAIADRLNVQPWQQFEKVVPETIRL      260

                                                            I
SchgrSAD      GNALVTQLIPACE-----GGAGLLGRLLAAGVSHDDARRLVVDLLLAAGDTTAYSTLWLL      304
LocmiSAD      GNALVTRLIPECA-----AGNGLLGRLLAAGVSHDDARRLVVDLLLAAGDTTAYSMLWLV      307
DromeSAD      GAAIIDHCIRVQEDQRRPHDEALYHRLQAADVPGDMIKRIFVDLVIAAGDTTAFSSQWAL      354
BommoSAD      AQKIVYEMLHTKD-----AGDGLVKRLKDENMSDELITRIVADFVIAAGDTTAYTSLWIL      319
AnogaSAD      ATAIIEFGIANAQ-----TRDGLLD-LMMQKLDKPLMMRIFIDFIIAAGDTTAFATVWAL      314

                                                   K
SchgrSAD      WLLAKHPSVQEQLHRELTRAP---AEEVARLPLARGAMREALRLFPVAPFLSRYLPEDAT      361
LocmiSAD      LLLAKHPSVQEELHGALSQPAAAEPEAAARLPLARGVIREALRLYPVAPFLCRYLPEDAD      367
DromeSAD      FALSKEPRLQQRLAKER---------ATNDSRLMHGLIKESLRLYPVAPFIGRYLPQDAQ      405
BommoSAD      FLLSNNTEILTEMN--------------DNDQYVKNVVKEAMRLYPVAPFLTRILPKQCV      365
AnogaSAD      YLLASNANLQQSVRQDVLDSN------TLECGAVKGVVRETLRLYPIAPFIGRFVEHESV      368

                                   PERF
SchgrSAD      IGGYHVPAGQLVVLSLYTSGRSEDYFPQAGRFWPQRWLRDPGGGYRGV-AASHASLPFAM      420
LocmiSAD      IGGYRVSAGQLVVLSLYTSGRSEANFPQAGRFWPQRWLRDSAGAYRGV-TASHASLPFAM      426
DromeSAD      LGGHFIEKDTMVLLSLYTAGRDPSHFEQPERVLPERWCIG---ETEQV-HKSHGSLPFAI      461
BommoSAD      LGPYLLEEGTPVIASIYTSGRDEQNFSKADQFLPYRWDRNDQRKKDLVNHVPSATLPFAF      425
AnogaSAD      FGAYALPKDTLVLLSLYSAGRDERFFAEPEAFNPYRWQRTNAAESSTG-RTPSASLPFAI      427

              heme
SchgrSAD      GARSCVGQKLAETQMALIVAEITKRFRLELADDE---DPEMVLRLVSVPSKPIRLRPVPR      477
LocmiSAD      GARSCVGQKLAETQMALLIAEITKRFRLELADGAEP-EPEMVLRLVSVPSRPVRVRPLPR      485
DromeSAD      GQRSCIGRRVALKQLHSLLGRCAAQFEMSCLNE--M-PVDSVLRMVTVPDRTLRLALRPR      518
BommoSAD      GARSCIGKKMAMLQMTELISQIVKNFDLKSMNNS---DVDAVTSQVLVPNKDIKVLILPR      482
AnogaSAD      GARSCIGQKIAQLQMHYLLSMILTKFDLTLAEADQQDAIKPILKMITVPSAPVKLCLKPC      487

SchgrSAD      H-------------      478
LocmiSAD      L-------------      486
DromeSAD      TE------------      520
BommoSAD      SISK----------      486
AnogaSAD      SAQPERQAVAQMQD      501
```

Supplementary Figure S4. Multiple sequence alignment of the *Schgr*SAD (MZ780957) with other identified and predicted SAD proteins from *Locusta migratoria* (*Locmi*SAD, AVL92827), *Drosophila melanogaster* (*Drome*SAD,

AAF54711), *Bombyx mori* (*Bommo*SAD, NP_001106224) and *Anopheles gambiae* (*Anoga*SAD, ABU42524). The multiple sequence alignment was made using the M-Coffee web server in the default settings (http://tcoffee.crg.cat/apps/tcoffee/do:mcoffee). The degree of conservation was highlighted using Boxshade and is indicated as follows: residues highlighted in black: complete; residues highlighted in dark grey: strong; residues highlighted in light grey: weak. Several key motifs for CYP450 enzymes are shown: P/G rich domain, helix-C, helix-I, helix-K, PERF-motif and the heme-binding domain.

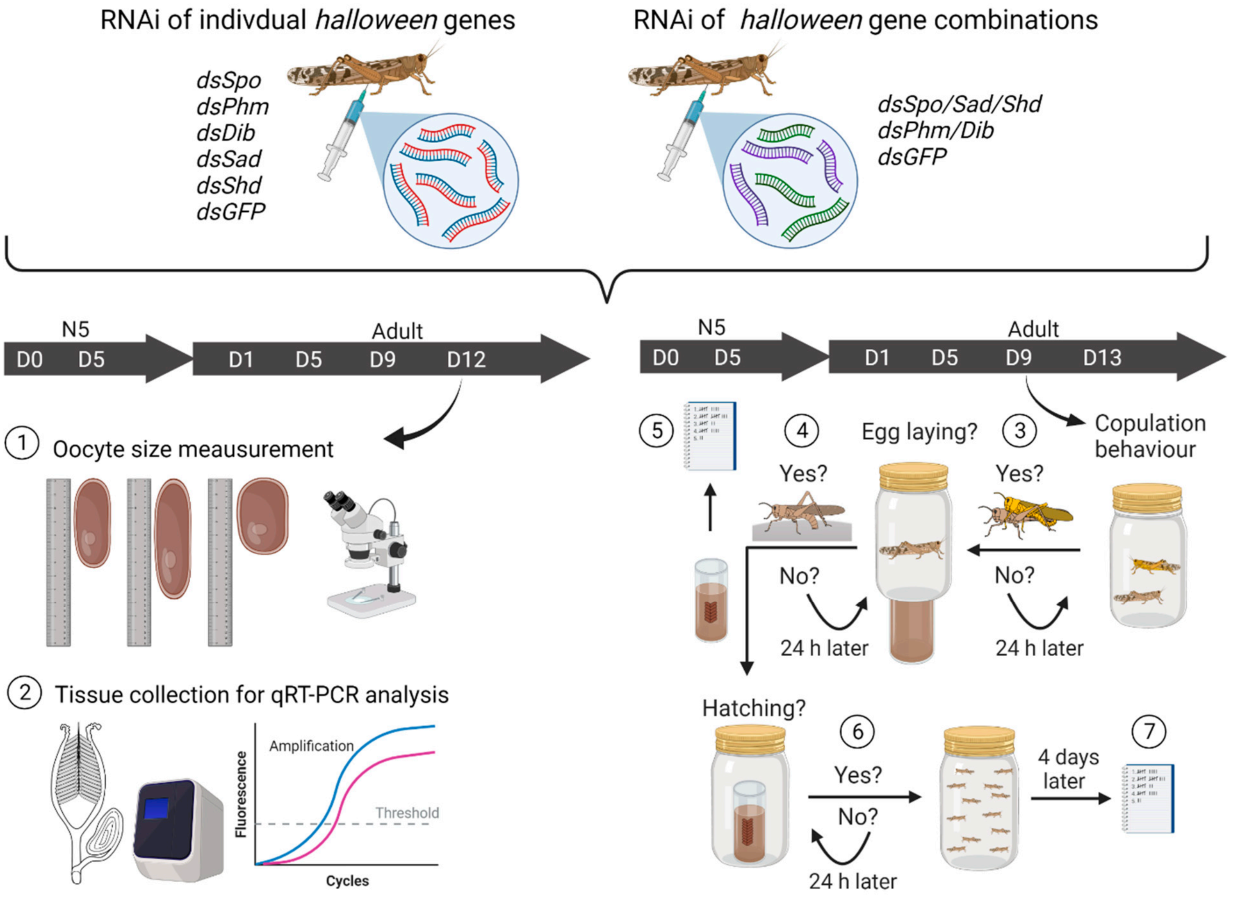

Supplementary Figure S5. Graphical representation of the performed experiments. Two different types of injections were performed. First, each *Halloween* gene was downregulated separately by injecting dsRNA targeting one of the genes of interest (*dsSpo*, *dsPhm*, *dsDib*, *dsSad*, *dsShd* and *dsGFP*). Second, *Halloween* genes *SchgrSpo*, *SchgrSad* and *SchgrShd*, as well as *SchgrPhm* and *SchgrDib*, were downregulated together by injecting a combination of different dsRNA molecules (*dsSpo*/*Sad*/*Shd* and *dsPhm*/*Dib*). Female locusts were injected on day 0 and day 5 of the 5th nymphal stage and day 1, day 5 and day 9 of the adult stage. A first group of female locusts was dissected on day 12 of the adult stage for measuring the size of basal oocytes and for RNA sample preparations. From each individual female, the length and width of five basal oocytes were measured (Observation ①). An ovary was collected to determine transcript levels of genes of interest by means of qRT-PCR (Observation ②). A second group of female locusts was used for an observational analysis of their copulation behavior, fecundity and fertility. From day 9 of the adult stage, female locusts were joined with a virgin male for two hours during which occurrence of copulation was monitored. If copulation did not occur in these two hours, the male was removed from the container and the experiment was repeated the following day. If copulation did occur, the male and female were allowed to stay together for the following 22 hours (Observation ③). Afterwards, the male was removed, and an egg pot was placed under the individual container. Egg pots were controlled daily for the presence of deposited eggs (Observation ④), and if eggs were laid their numbers were counted (Observation ⑤). Lastly, all pots were controlled daily for the appearance of hatchlings (Observation ⑥) and the number of hatchlings was counted four days after hatching (Observation ⑦). The second group of female locusts had received an extra dsRNA injection on day 13 of the adult stage. (This scheme was made using Biorender®)

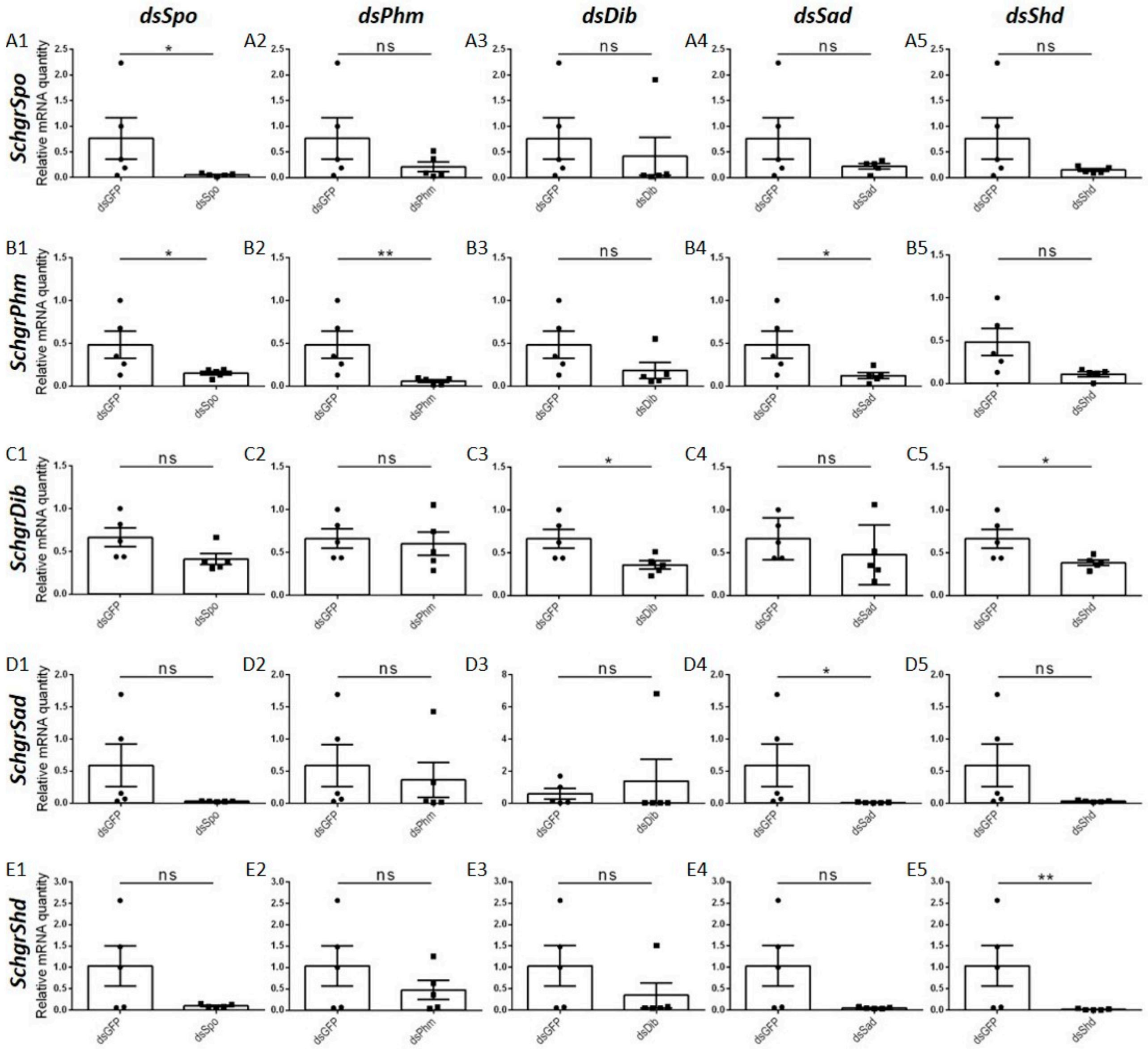

Supplementary Figure S6. Transcript levels of five *Halloween* genes (*SchgrSpo*, *SchgrPhm*, *SchgrDib*, *SchgrSad* and *SchgrShd*) in the ovaries of *dsSpo-*, *dsPhm-*, *dsDib-*, *dsSad-* or *dsShd*-injected adult female locusts. (A1-5) Relative *SchgrSpo* transcript levels measured in the ovaries of *dsSpo-*, *dsPhm-*, *dsDib-*, *dsSad-* and *dsShd-treated* females, respectively. **(B1-5)** Relative *SchgrPhm* transcript levels measured in the ovaries of *dsSpo-*, *dsPhm-*, *dsDib-*, *dsSad-* and *dsShd-treated* females, respectively. **(C1-5)** Relative *SchgrDib* transcript levels measured in the ovaries of *dsSpo-*, *dsPhm-*, *dsDib-*, *dsSad-* and *dsShd-treated* females, respectively. **(D1-5)** Relative *SchgrSad* transcript levels measured in the ovaries of *dsSpo-*, *dsPhm-*, *dsDib-*, *dsSad-* and *dsShd-treated* females, respectively. **(E1-5)** Relative *SchgrShd* transcript levels measured in the ovaries of *dsSpo-*, *dsPhm-*, *dsDib-*, *dsSad-* and *dsShd* -treated females, respectively. All relative transcript levels were measured in the ovaries of female locusts twelve days after the final molt using qRT-PCR. The data are presented as mean ± S.E.M of five independent pools of three locusts, run in duplicate and normalized to *glyceraldehyde 3-phosphate dehydrogenase* (*GAPDH*) and *CG13220* transcript levels; each dot represents one data point. Significant differences in comparison with *dsGFP*-injected females are indicated by (an) asterisk(s) (** $p < 0.01$ and * $p < 0.5$; two-sided Unpaired t-test on log-transformed data including the Welch's correction for comparisons of the *SchgrSpo* transcript levels in *dsShd-* and *dsGFP-treated* females (A5), *SchgrPhm* transcript levels in *dsShd-* and *dsGFP-treated* females (B5), *SchgrSad* transcript levels in *dsSpo-* (D1), *dsSad-* (D4), *dsShd-* (D5) and *dsGFP-treated* females and *SchgrShd* transcript levels in *dsSpo-* (E1), *dsShd-* (E4) and *dsGFP-treated* females).

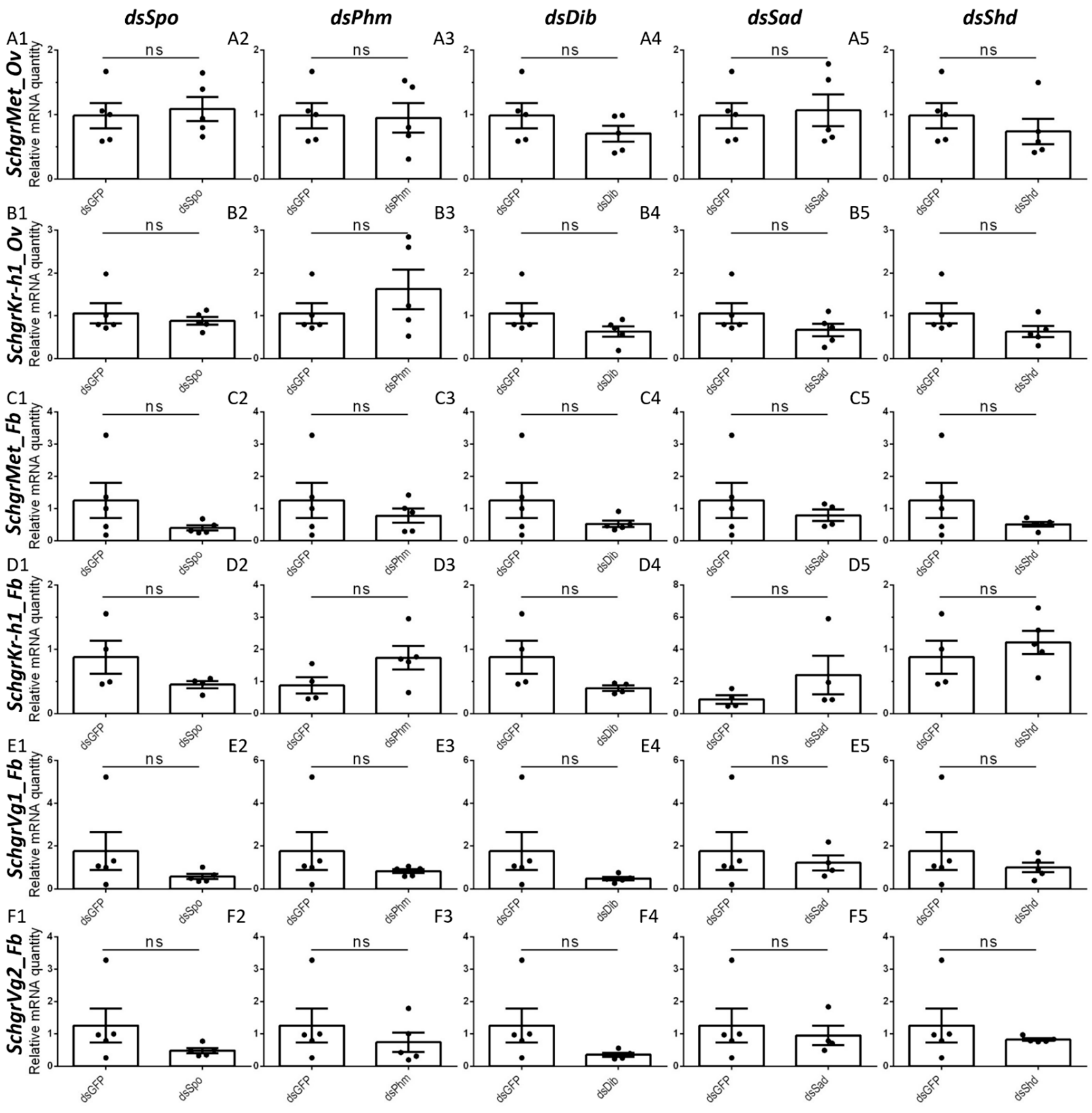

Supplementary Figure S7. Transcript levels of genes involved in vitellogenin (Vg) synthesis in the fat body and JH signalling in the fat body and ovaries of *dsSpo-*, *dsPhm-*, *dsDib-*, *dsSad-* or *dsShd*-injected adult female locusts. (A1-5) Relative *SchgrMet* transcript levels measured in the ovaries of *dsSpo-*, *dsPhm-*, *dsDib-*, *dsSad-* or *dsShd*-treated females, respectively. **(B1-5)** Relative *SchgrKr-h1* transcript levels measured in the ovaries of *dsSpo-*, *dsPhm-*, *dsDib-*, *dsSad-* or *dsShd-*treated females, respectively. **(C1-5)** Relative *SchgrMet* transcript levels measured in the fat body of *dsSpo-*, *dsPhm-*, *dsDib-*, *dsSad-* or *dsShd*-treated females, respectively. **(D1-5)** Relative *SchgrKr-h1* transcript levels measured in the fat body of *dsSpo-*, *dsPhm-*, *dsDib-*, *dsSad-* or *dsShd*-treated females, respectively. **(E1-5)** Relative *SchgrVg1* transcript levels measured in the fat body of *dsSpo-*, *dsPhm-*, *dsDib-*, *dsSad-* or *dsShd*-treated females, respectively. **(F1-5)** Relative *SchgrVg2* transcript levels measured in the fat body of *dsSpo-*, *dsPhm-*, *dsDib-*, *dsSad-* or *dsShd*-treated females, respectively. All relative transcript levels were measured in the fat body or ovaries of female locusts twelve days after the final molt using qRT-PCR. The data represent the mean ± S.E.M of five independent pools of three locusts, run in duplicate and normalized to *CG13220* and *β-actin* transcript levels; each dot represents one data point. No significant differences were observed in comparison with *dsGFP*-injected females (two-sided Unpaired t-test on log-transformed data including the Welsch's correction for the comparison of *SchgrVg1* transcript levels of *dsPhm-* and *dsGFP*-treated females (E2) and of *SchgrVg2* transcript levels in *dsShd-* and *dsGFP*-treated females (F5)).

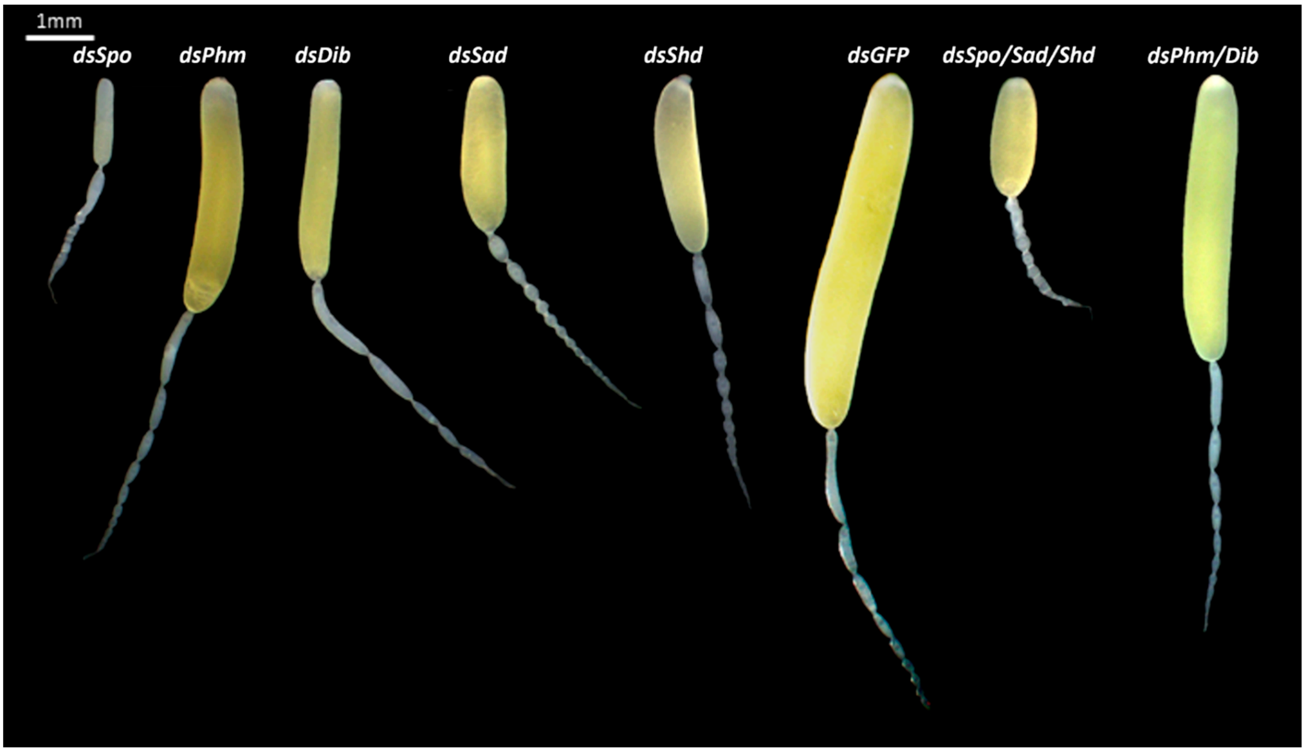

Supplementary Figure S8. Representative ovarioles of *dsSpo-*, *dsPhm-*, *dsDib-*, *dsSad-*, *dsShd-*, *dsGFP-*, *dsSpo/Sad/Shd-* and *dsPhm/Dib*-injected females. *dsSpo* basal oocyte: length = 1.3 mm – width = 0.3 mm; *dsPhm* basal oocyte: length = 3.5 mm – width = 0.6 mm; *dsDib* basal oocyte: length = 3.0 mm – width = 0.5 mm; *dsSad* basal oocyte: length = 2.3 mm – width = 0.7 mm; *dsShd* basal oocyte: length = 2.6 mm – width = 0.6 mm; *dsGFP* basal oocyte: length = 5.2 mm – width = 0.9 mm; *dsSpo/Sad/Shd* basal oocyte: length = 1.8 mm – width = 0.7 mm; *dsPhm/Dib* basal oocyte: length = 4.2 mm – width = 0.7 mm. Scale bar = 1 mm.

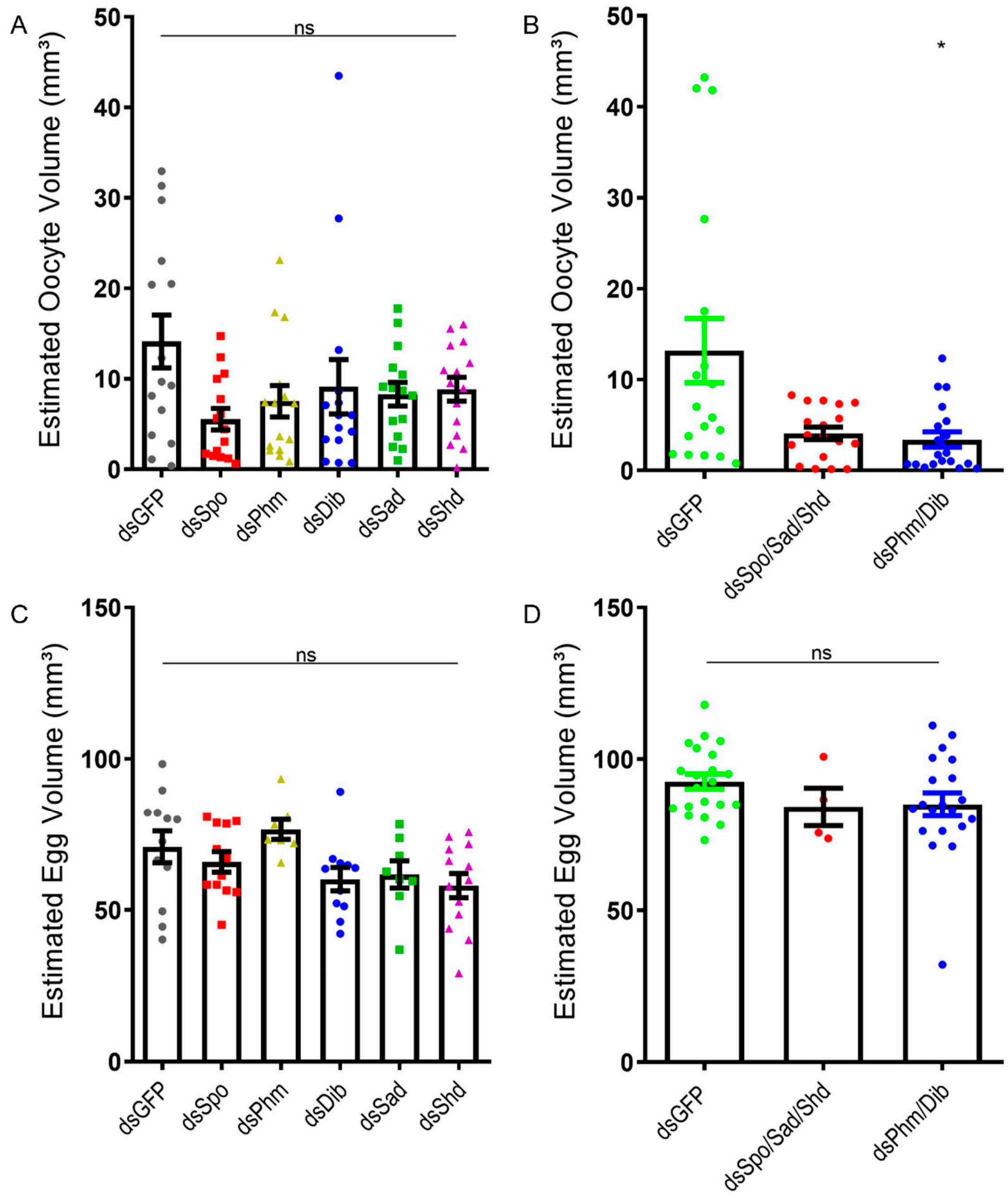

Supplementary Figure S9. Estimated volume (calculated based on length and width measurements) of oocytes and eggs derived from *dsSpo-*, *dsPhm-*, *dsDib-*, *dsSad-*, *dsShd-*, *dsGFP-*, *dsSpo/Sad/Shd-* and *dsPhm/Dib*-injected females. **(A)** The volume of five basal oocytes per *dsSpo-*, *dsPhm-*, *dsDib-*, *dsSad-*, *dsShd-* or *dsGFP*-injected female locust (n = 15 for all conditions) is represented as mean ± S.E.M.; each dot represents one data point (Kruskal-Wallis test with Dunn's multiple comparison test) **(B)** The volume of five basal oocytes per *dsSpo/Sad/Shd-*, *dsPhm/Dib-* or *dsGFP*-injected female locust (n = 15 for all conditions) is represented as mean ± S.E.M.; each dot represents one data point. Significant differences are indicated by an asterisk (* $p < 0.05$; Kruskal-Wallis test with Dunn's Multiple Comparison Test). **(C)** The volume of 20 eggs per *dsSpo-* (n = 12), *dsPhm-* (n = 7), *dsDib-* (n = 11), *dsSad-* (n = 8), *dsShd-* (n = 13) or *dsGFP*-injected (n = 12) female locust is represented as mean ± S.E.M;. each dot represents one data point (One-way ANOVA test with Dunnett's multiple comparison test) **(D)** The volume of seven eggs per *dsSpo/Sad/Shd-* (n = 4), *dsPhm/Dib-* (n = 24) or *dsGFP*-injected (n = 23) female locust is represented as mean ± S.E.M;. each dot represents one data point (Kruskal-Wallis test with Dunn's multiple comparison test).

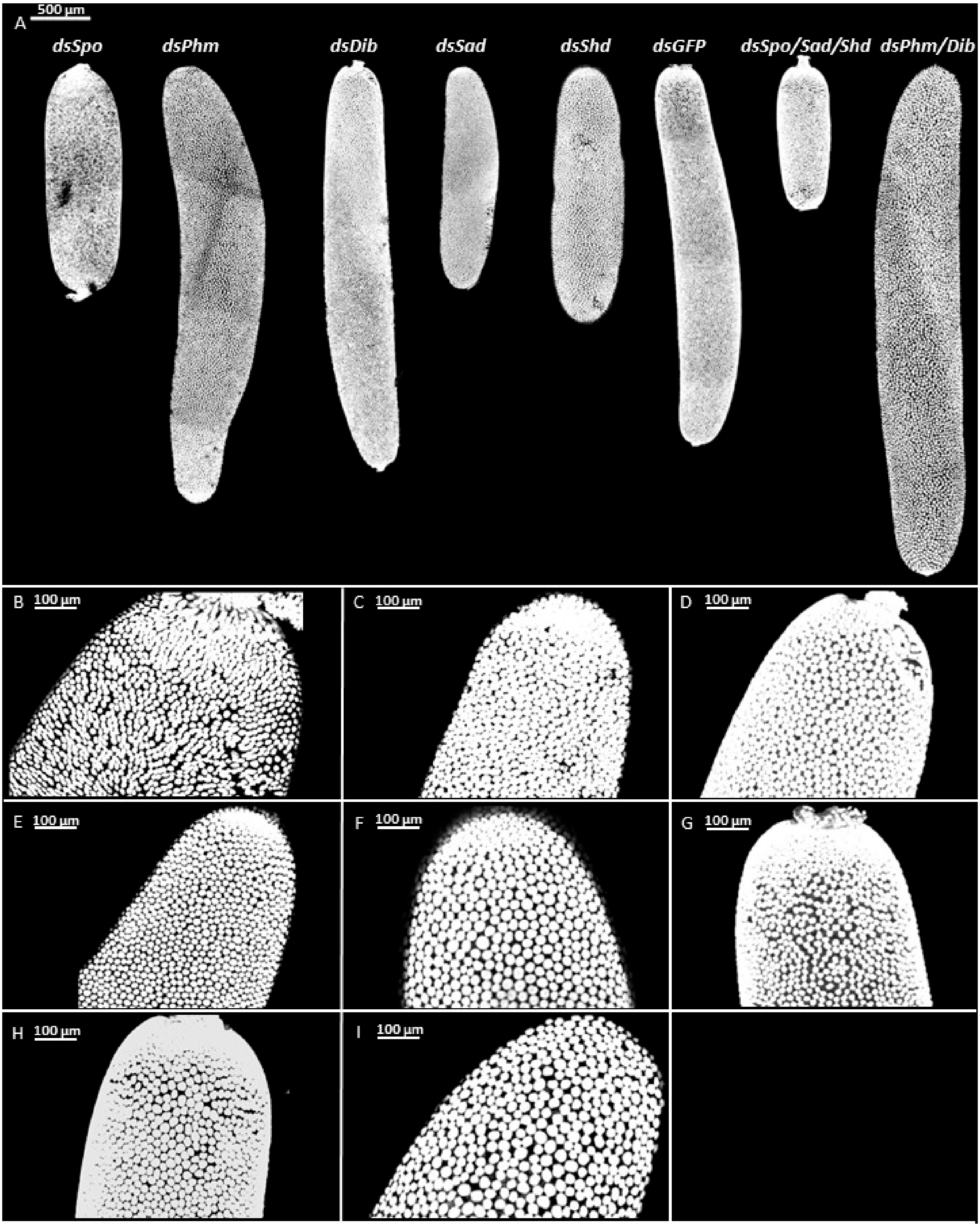

Supplementary Figure S10. DAPI-stained nuclei of follicular epithelial cells surrounding the basal oocytes in representative ovarioles derived from *dsSpo-*, *dsPhm-*, *dsDib-*, *dsSad-*, *dsShd-*, *dsGFP-*, *dsSpo/Sad/Shd-* and *dsPhm/Dib*-injected females. (A) *dsSpo* basal oocyte: length = 1.9 mm, width = 0.6 mm; *dsPhm* basal oocyte: length = 3.6 mm, width = 0.7 mm; *dsDib* basal oocyte: length = 3.4 mm, width = 0.6 mm; *dsSad* basal oocyte: length = 1.9 mm, width = 0.5 mm; *dsShd* basal oocyte: length = 2.1 mm, width = 0.6 mm; *dsGFP* basal oocyte: length = 3.1 mm, width = 0.5 mm; *dsSpo/Sad/Shd* basal oocyte: length = 1.2 mm, width = 0.4 mm; *dsPhm/Dib* basal

oocyte: length = 4.1 mm, width = 0.7 mm. Scale bar = 500 µm. **(B-I)** Zoomed in pictures of the distal end of the ovarioles of *dsSpo-* **(B),** *dsPhm-* **(C)**, *dsDib-* **(D)**, *dsSad-* **(E)**, *dsShd-* **(F)**, *dsGFP-* **(G)**, *dsSpo/Sad/Shd-* **(H)** and *dsPhm/Dib-* **(I)** treated females. Scale bar = 100 µm.

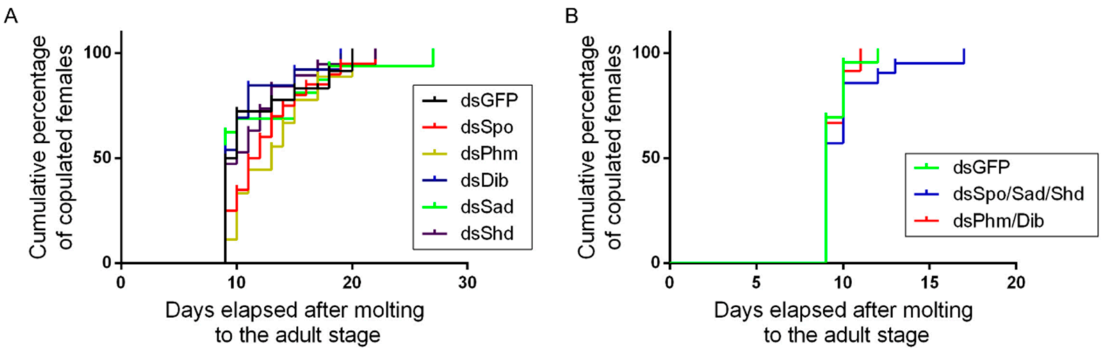

Supplementary Figure S11. Observations of mating by *dsSpo-*, *dsPhm-*, *dsDib-*, *dsSad-*, *dsShd-*, *dsGFP-*, *dsSpo/Sad/Shd-* and *dsPhm/Dib*-injected females. (A) Cumulative percentage of copulated *dsSpo-*, *dsPhm-*, *dsDib-*, *dsSad-*, *dsShd-* and *dsGFP*-injected female locusts is presented. The numbers of female locusts which copulated (per treatment condition) are: *dsSpo* N = 19/20 (95%), *dsPhm* N = 9/9 (100%), *dsDib* N = 13/13 (100%), *dsSad* N = 16/16 (100%), *dsShd* N = 19/19 (100%) and *dsGFP* N = 17/18 (95%). **(B)** Cumulative percentage of copulated *dsSpo/Sad/Shd-*, *dsPhm/Dib-* and *dsGFP*-injected female locusts is presented. The numbers of female locusts which copulated (per treatment condition) are: *dsSpo/Sad/Shd* N = 21, *dsPhm/Dib* N = 23 and *dsGFP* N = 24.

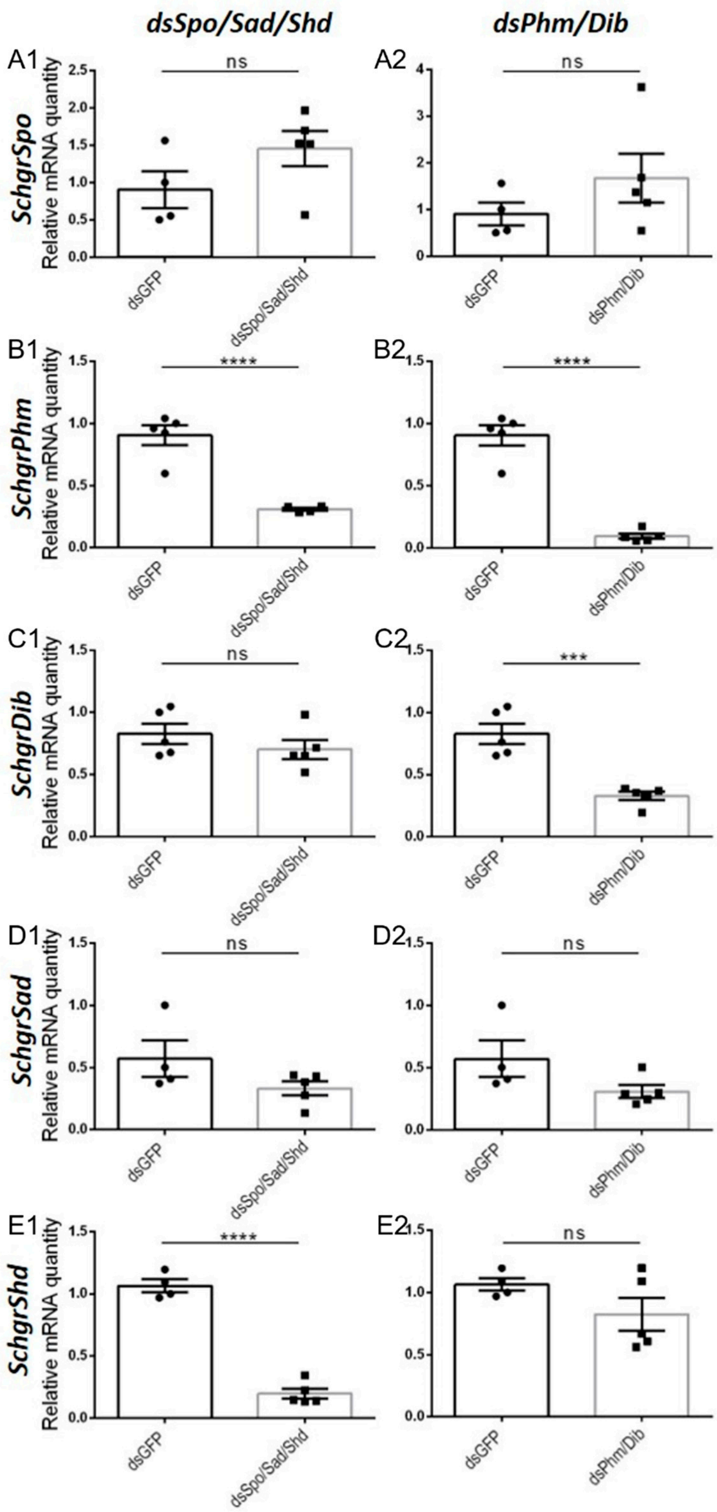

Supplementary Figure S12. Transcript levels of *Halloween* genes (*SchgrSpo, SchgrPhm, SchgrDib, SchgrSad* and *SchgrShd*) in ovaries of *dsSpo/Sad/Shd*- and *dsPhm/Dib*-injected adult female locusts. (A1-2) Relative *SchgrSpo* transcript levels measured in the ovaries of *dsSpo/Sad/Shd*- and *dsPhm/Dib*-treated females, respectively. **(B1-2)** Relative *SchgrPhm* transcript levels measured in the ovaries of *dsSpo/Sad/Shd*- and *dsPhm/Dib*-treated females, respectively. **(C1-2)** Relative *SchgrDib* transcript levels measured in the ovaries of *dsSpo/Sad/Shd*- and *dsPhm/Dib*-treated females, respectively. **(D1-2)** Relative *SchgrSad* transcript levels measured in the ovaries of *dsSpo/Sad/Shd*- and *dsPhm/Dib*-treated females, respectively. **(E1-2)** Relative *SchgrShd* transcript levels measured in the ovaries of *dsSpo/Sad/Shd*- and *dsPhm/Dib*-treated females, respectively. All relative transcript levels were measured in the ovaries of female locusts twelve days after the final moult using qRT-PCR. The data are presented as mean ± S.E.M of five independent pools of three locusts, run in duplicate and normalized to *EF1α* and *ribosomal protein 49* (*RP49*); each dot represents one data point. Significant differences in comparison

with *dsGFP*-injected females are indicated by asterisks (**** $p < 0.0001$ and *** $p < 0.001$; two-sided Unpaired *t*-test on log-transformed data including the Welch's correction for the comparison of *SchgrShd* transcript levels in *dsSpo*/*Sad*/*Shd*- and *dsGFP*-treated females (E1)).

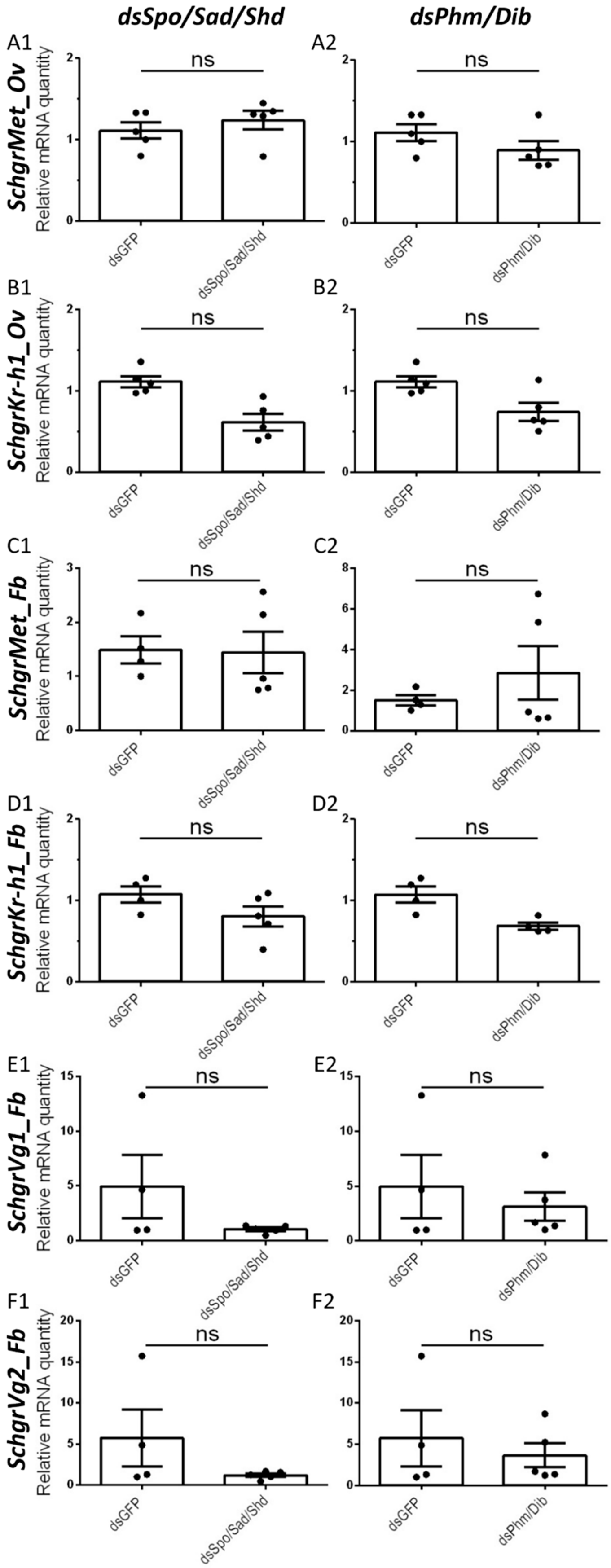

Supplementary Figure S13. Transcript levels of genes involved in Vg synthesis in the fat body and JH signalling in the fat body and ovaries of *dsSpo/Sad/Shd-* and *dsPhm/Dib*-injected adult female locusts. (A1-2) Relative *SchgrMet*

transcript levels measured in the ovaries of *dsSpo*/*Sad*/*Shd*- and *dsPhm*/*Dib*-treated females, respectively. **(B1-2)** Relative *SchgrKr-h1* transcript levels measured in the ovaries of *dsSpo*/*Sad*/*Shd*- and *dsPhm*/*Dib*-treated females, respectively **(C1-2)** Relative *SchgrMet* transcript levels measured in the fat body of *dsSpo*/*Sad*/*Shd*- and *dsPhm*/*Dib*-treated females, respectively. **(D1-2)** Relative *SchgrKr-h1* transcript levels measured in the fat body of *dsSpo*/*Sad*/*Shd*- and *dsPhm*/*Dib*-treated females, respectively. **(E1-2)** Relative *SchgrVg1* transcript levels measured in the fat body of *dsSpo*/*Sad*/*Shd*- and *dsPhm*/*Dib*-treated females, respectively. **(F1-2)** Relative *SchgrVg2* transcript levels measured in the fat body of *dsSpo*/*Sad*/*Shd*- and *dsPhm*/*Dib*-treated females, respectively. All relative transcript levels were measured in the ovaries of female locusts twelve days after the final moult using qRT-PCR. The data represent the mean ± S.E.M of five independent pools of three locusts, run in duplicate and normalized to *RP49* and *EF1α* transcript levels in the fat body and ovaries; each dot represents one data point. No significant differences were observed in comparison with *dsGFP*-injected females (two-sided Unpaired *t*-test on log-transformed data including the Welsch's correction for the comparison of *SchgrKr-h1* transcript levels in the fat body of *dsPhm*/*Dib*- and *dsGFP*-treated females (D2)).
